# Microwave-Assisted Synthesis and Characterization of Novel 1,3,4-Oxadiazole Derivatives and Evaluation of In Vitro Antimycobacterial Activity

**DOI:** 10.7759/cureus.69679

**Published:** 2024-09-18

**Authors:** Mahalakshmi Devaraji, Punniyakoti V Thanikachalam

**Affiliations:** 1 Department of Pharmaceutical Chemistry, Saveetha College of Pharmacy, Saveetha Institute of Medical and Technical Sciences, Chennai, IND

**Keywords:** admet prediction, anti-tubercular activity, microwave irradiation, molecular docking, mycobacterium tuberculosis, oxadiazole derivatives

## Abstract

Objective

The study's goal was to come up with and make a new group of 1,3,4-oxadiazole derivatives (3a-3e) and test how well they could kill *Mycobacterium tuberculosis* (Mtb) H37Rv strain. Additionally, molecular docking and pharmacokinetic properties were analyzed using computational software to identify potential inhibitors, followed by in vitro antimycobacterial assays.

Methods

A group of 1,3,4-oxadiazoles was prepared by reacting acyl hydrazides with alanine, an N-protected α-amino acid, and a small amount of POCl_3_. This was carried out under microwave treatment. The structural characterization of the newly synthesized compounds was performed using infrared (IR) spectroscopy, nuclear magnetic resonance (NMR) spectroscopy, and mass spectrometry. The in vitro antimycobacterial activity of the 1,3,4-oxadiazole derivatives (3a-3e) was assessed using the microplate Alamar Blue assay against the Mtb H37Rv strain. The synthesized compounds were subjected to molecular docking investigations in order to gain insights into their interaction mechanisms with the mycobacterial enzyme InhA (enoyl-acyl carrier protein reductase). Computational analysis of pharmacokinetic properties was performed to predict the oral bioavailability and drug-likeness of the compounds.

Results

All synthesized compounds inhibited the growth of Mtbat concentrations of 50 and 100 μg/mL. At a concentration of 50 μg/mL, compounds 3c and 3d exhibited the most prominent antimycobacterial action. Molecular docking results revealed that compound 3d exhibited the highest binding energy interaction with the InhA enzyme (-9.1 kcal/mol). Pharmacokinetic predictions indicated that all compounds possess favorable drug-like properties suitable for oral administration.

Conclusion

This study successfully synthesized a novel series of oxadiazole derivatives (3a-3e) using a microwave-assisted method with high yields. The synthesized compounds demonstrated significant antimycobacterial activity, particularly compounds 3c and 3d. Molecular docking and pharmacokinetic analyses further confirmed the potential of these compounds as promising leads for the development of anti-tubercular agents.

## Introduction

Tuberculosis (TB) is a major worldwide cause of mortality and is the primary cause of death resulting from an infectious agent, specifically Mycobacterium TB (Mtb). Mtb predominantly targets the respiratory system, but it can also infiltrate other organs, such as the brain, spine, and kidney, leading to the development of extra-pulmonary TB [1]. People with pulmonary TB can transmit the disease through the air when they cough, spit, or sneeze. In 2019, the worldwide prevalence of TB was approximately 10 million cases. Among these, males accounted for 5.6 million cases, females for 3.2 million cases, and children for 1.2 million cases. TB is a disease that affects individuals of all age groups and is widespread in all countries. However, TB is both curable and avoidable [2]. The World Health Organization (WHO) created the "End TB" initiative in 2035 to eliminate TB [3]. India's ambitious goal to eliminate TB by 2025 continues to expand, even though India has the highest number of TB cases. One of the main challenges in achieving global targets for TB elimination is developing novel, improved, or groundbreaking antimycobacterial medications to combat resistance [4]. The approved drugs for treating TB include isoniazid, streptomycin, ethambutol, moxifloxacin, ciprofloxacin, rifampicin, ofloxacin, pyrazinamide, and levofloxacin. These medications are utilized as a primary and secondary treatment for TB. However, the data indicate that Mtb does not respond favorably to the current therapies. In addition, a significant number of these medications have had adverse effects on both morbidity and mortality rates [5]. As a result of the emergence of multi-drug resistant (MDR) and extensive-drug-resistant TB, the prospects for patients have recently worsened, highlighting the need for the creation of novel treatments that are both effective and safe.

Important heterocyclic compounds, 1,3,4-oxadiazoles and their derivatives, have generated much interest in polymer and material science and pharmaceutical and insecticide chemistry. These derivative compounds have been discovered to possess a wide range of biological activities, including antibacterial [6], anti-inflammatory [7], anti-fungal [8], analgesic [9], antimalarial [10], antimicrobial [11], and other biological properties [12]. As a result, there has been significant interest in creating compounds that contain this specific heterocyclic structure, and a diverse range of techniques have been employed to construct them.

The prevailing method for synthesizing these compounds is through the dehydrative cyclization of diacyl hydrazides, typically employing potent acidic reagents, including phosphorus pentoxide, sulfuric acid, thionyl chloride, and phosphorus oxychloride [13]. Recently, researchers have utilized 1,3,4-oxadiazoles to create novel peptidomimetics. Sureshbabu et al. elucidated the procedure for generating 1,3,4-oxadiazole peptidomimetics by synthesizing diacyl hydrazines derived from amino acids [14,15]. Conversely, amino acids have become crucial components for creating many molecules. Furthermore, there has been a growing recognition of the significance of amino acids in their biological and therapeutic activities, such as antioxidant, anticancer, antibacterial, and antiviral characteristics.

The present study focused on producing a novel group of oxadiazole compounds and their efficacy against mycobacterial infections. In addition, molecular docking is used to forecast the interaction between a ligand and a specific protein and identify where they bind together. It helps develop a potent medicinal agent targeting the protein of interest.

## Materials and methods

Chemicals and reagents 

The solvents and chemical substances used were purchased from commercial vendors. We utilized 3 × 15 cm thin-layer chromatography (TLC) plates treated with silica gel G for analytical TLC to examine the completion of reactions and measure the coefficient of retardation. The iodine chamber was used to identify the TLC spots. The melting temperatures of synthesized derivatives were measured using a digital melting point instrument (Shimadzu AUX-220, Shimadzu Corporation, Kyoto, Japan) and were recorded without any corrections. The double-beam UV-visible 1800 Shimadzu spectrophotometer (Shimadzhu-1700, Shimadzu Medical (India) Pvt. Ltd., Mumbai, India) was used to find the maximum wavelength (𝜆max). The infrared (IR) spectra were obtained using a Fourier transform infrared spectroscopy (FTIR) spectroscopy (Shimadzu Corporation, Kyoto, Japan) employing the potassium bromide (KBr) technique. The proton nuclear magnetic resonance (^1^H NMR) spectra were acquired using a Bruker Avance III 400 MHz spectrometer (Bruker Corporation, Billerica, MA) with a frequency of 400 MHz. The Perkin Elmer Clarus 680 gas chromatograph-mass spectrometer (GC-MS) (PerkinElmer, Inc., Waltham, MA) was utilized to obtain mass spectra. The software ChemDraw Ultra (PerkinElmer, Inc., Waltham, MA) was used to conduct structural similarity analysis. The molecular docking research utilized AutoDock Vina (Scripps Research, San Diego, CA) and Discovery Studio Visualizer (BIOVIA, San Diego, CA) tools to visualize the protein-ligand interaction.

Chemical synthesis

Synthesis of Acid Hydrazides (2a-2e)

Aromatic acids (0.01 mol) are treated with ethanol (60 mL) and concentrated sulfuric acid (1.5 mL) and kept in a microwave oven for 10 minutes at 100 watts. Then, the contents were cooled and poured into a beaker containing 200 mL of crushed ice, and then the mixture was neutralized with ammonia. Subsequently, it was extracted using five aliquots (5 × 25 mL) of diethyl ether as the solvent. The mixture of ether extract was dehydrated using anhydrous potassium carbonate to obtain ester-substituted ethyl benzoate derivatives (1a-e), with a yield of 95%. In a beaker, 0.1 mol of aromatic ester, 0.15 mol of hydrazine hydrate, and 10 mL of ethanol were placed under heating in a 100-watt microwave oven for six minutes. It was then put in an ice bath to cool down. The acid hydrazides were left, filtered, and dried (2a-e), yielding 92%.

Synthesis of N-Protected Amino Acids: Benzoylation of Alanine

Alanine (0.25 mol) was dissolved in 0.25 mol of 4N sodium hydroxide and immersed in an ice bath till the temperature dropped to 3°C. Benzoyl chloride (0.5 mol) was added dropwise with stirring, and the reaction temperature should remain below 20°C). Stirring continued for one hour. Sodium benzoate (0.25 mol) and 0.5 mol of sodium chloride (0.5 mol) were added to the above content. A solution of 4 mol of hydrochloric acid (0.25 mol) was added to the reaction mixture while stirring to achieve neutralization. The mixture was then maintained at a temperature of 5°C for a duration of 12 hours. The precipitate of benzoyl alanine separated, and it was filtered and dried. The yield was found to be 96%.

General Procedure for Synthesis of N-[1-(5-Phenyl-1,3, 4-Oxadiazol-2-Yl) Ethyl] Benzamide (3a-3e)

Acid hydrazides (1 mol) and N-protected amino acids (0.015 mol) were dissolved in phosphorous oxychloride (5 mL) and then placed in a microwave (100 W) for 10 minutes. Then, the mixture was poured into crushed ice after it had cooled down. The solid residue separated was then filtered and dried. The completion of the reaction was monitored by TLC using petroleum ether:ethylacetate (8:2) as a mobile phase. The compounds were recrystallized using methanol [16,17].

N-{1-[5-(4-chlorophenyl)-1,3,4-oxadiazol-2-yl]ethyl}benzamide (3a): The yield of the compound is 85%, and it is a light yellow solid with a melting point range of 210-215°C. The IR spectrum (measured in cm^-1^) showed peaks at 3336.85 (nitrogen-hydrogen (NH) symmetrical stretching), 3032.10 (aromatic carbon-hydrogen (CH) stretching), 2937.59 (aliphatic CH stretching), 1691.57 (C=O stretching), 1598.99 (C=N stretching), 1255.66 (asymmetric C-O-C stretching), 1176.58 (CH bending), 1018.41 (symmetric C-O-C stretching), 941.26, 846.75 (CH bending), and 719.45 (carbon-chlorine (C-Cl) stretching). The ^1^H NMR spectrum (measured in ppm) in dimethyl sulfoxide (DMSO) at 400 MHz showed peaks at δ 3.93 (d, 3H, J = 6 Hz), 7.46-8.33 (m, 9H), 8.82-8.85 (ddd, 2H, J = 12 Hz). GC-MS (electrospray ionization (ESI)): calc. for C_17_H_14_ClN_3_O_2_ [M+H]+: 327.76, with an estimated value of 328.08.

N-{1-[5-(4-methylphenyl)-1,3,4-oxadiazol-2-yl]ethyl}benzamide (3b): The compound was obtained with a yield of 82% as a yellow solid. Its melting point is in the range of 213-215°C. The IR spectrum (measured in cm^-1^) showed peaks at 3338.78 (corresponding to NH symmetrical stretching), 3066.82 (CH aromatic stretching), 2937.59 (CH aliphatic stretching), 1658.78 (C=O stretching), 1554.63 (C=N stretching), 1249.87 (C-O-C asymmetric stretching), 1176.58 (CH bending), 1028.06 (C-O-C symmetric stretching), 943.19, and 848.68 (CH bending). The proton NMR spectrum (measured in ppm) in DMSO at 400 MHz showed peaks at δ 1.3 (s, 3H), 3.94 (d, 3H, J = 5.6 Hz), 6.91-7.89 (m, 9H), 8.81-8.85 (ddd, 2H, J = 12 Hz). GC-MS (ESI): calc. for C_17_H_15_N_3_O_3_ [M+H]+: 307.34, with an estimated value of 308.11.

N-{1-[5-(4-hydroxyphenyl)-1,3,4-oxadiazol-2-yl]ethyl}benzamide (3c): The compound was obtained with a yield of 89 % as a yellow solid. It has a melting point range of 215-220°C. The IR spectrum (measured in cm^-1^) showed peaks at 3336.85 (corresponding to NH symmetrical stretching), 3217.27 (OH symmetrical stretching), 3061.03 (aromatic CH stretching), 2937.59 (aliphatic CH stretching), 1695.43 (C=O stretching), 1552.70 (C=N stretching), 1255.66 (asymmetric stretching of C-O-C), 1176.58 (CH bending), 1028.06 (symmetric stretching of C-O-C), and 941.26 and 846.75 (CH bending). The proton NMR spectrum (measured in ppm) in DMSO at 400 MHz shows signals at δ 3.93 (d, 3H, J = 6 Hz), 7.47-8.34 (m, 9H), 8.82-8.85 (ddd, 2H, J = 11.6 Hz). GC-MS (ESI): calc. for C_17_H_15_N_3_O_3_ [M+H]+: 309.31, with an estimated value of 310.11.

N-{1-[5-(4-aminophenyl)-1,3,4-oxadiazol-2-yl]ethyl}benzamide (3d): The yield of the compound was 86%, and it appeared as a yellow solid. The melting point range was 220-225°C. The IR spectrum (measured in cm^-1^) showed peaks at 3336.85 (NH symmetrical stretching), 3062.96 (aromatic CH stretching), 1689.64 (C=O stretching), 1554.63 (C=N stretching), 1294.24 (asymmetric C-O-C stretching), 1178.51 (CH bending), 1078.21 (symmetric C-O-C stretching), 941.26, and 850.61 (CH bending). The ^1^H NMR spectrum (recorded in DMSO at 400 MHz) showed peaks at δ ^1^H NMR: δ 3.53 (d, 3H, J = 5.8 Hz), 5.29-7.64 (m, 9H), 9.94 (s, 2H). GC-MS (ESI): calc. for C_17_H_16_N_4_O_2_ [M+H]+: 308.33, with an estimated value of 309.13.

N-{1-[5-(4-nitrophenyl)-1,3,4-oxadiazol-2-yl]ethyl}benzamide (3e): The yield of the compound was 92%, and it appeared as a yellow solid. The melting point range was 210-215°C. The IR spectrum (measured in cm^-1^ using KBr) showed peaks at 3340.71 (corresponding to NH symmetrical stretching), 3034.03 (CH aromatic stretching), 2904.80 (CH aliphatic stretching), 1602.85 (C=O stretching), 1552.70 (C=N stretching), 1394.53 (N-O symmetrical stretching), 1244.09 (C-O-C asymmetrical stretching), 1176.58 (CH bending), 1018.41 (C-O-C symmetrical stretching), 962.48, and 846.75 (CH bending). The proton NMR spectrum (measured in DMSO at 400 MHz) showed peaks at δ 3.85-3.93 (d, 3H, J = 5.7 Hz), 6.89-7.93 (m, 9H), 8.86 (s, 2H). GC-MS (ESI): calc. for C_17_H_14_N_4_O_4_ [M+H]+: 338.31, with an estimated value of 339.10.

Computational study

Computational approaches are used in in silico investigations to examine and analyze biological properties. Computational studies utilize algorithms, computational models, or statistical frameworks to gain knowledge about complex systems and make predictions. Molecular docking studies involve specialized computer analysis techniques that aim to anticipate the interaction affinity and configurations of small molecules (ligands) within the binding site of the target protein. Molecular docking (AutoDock Vina) is a software tool used to simulate and study the binding of a ligand and a protein, as well as assess their interactions and compatibility. These investigations are important in the field of drug discovery and design since they can help find new potential drugs by evaluating their ability to bind to a certain target protein [18].

Molecular docking study

Molecular docking is the computational simulation of the interaction between two molecules to predict their binding affinity and orientation. Molecular docking analysis is a frequently employed method for studying complex and protein interactions. This methodology can ascertain the optimal procedure for interacting with pharmaceuticals and biomolecules by forecasting the binding affinity between these compounds and protein molecules [19].

In this study, we employed molecular docking techniques to understand better the molecular interactions between small molecules, specifically 1,3,4-oxadiazole derivatives and specific protein targets, such as InhA inhibitors. InhA is a critical enzyme in the fatty acid synthesis pathway of Mtb, making it a prominent target for anti-TB drug development. This enzyme catalyzes the nicotinamide adenine dinucleotide (NADH)-dependent reduction of enoyl-acyl carrier protein reductase (enoyl-ACP), a crucial step in the fatty acid elongation process essential for mycobacterial cell wall synthesis [20,21]. This research aimed to determine or confirm the potential of these small molecules as anti-TB drugs.

Analysis of molecular docking: synthesis of protein-ligand complex

The protein from RCSB was produced using the Discovery Studio software and stored in the database's PDB file format. The unprepared protein containing water molecules, ligands, and heteroatoms was removed from the protein using Discovery Studio software. The receptor and ligand were successfully loaded into the AutoDock computerized docking software to carry out the docking simulation [22].

Molecular docking techniques: detection of active regions within the protein

**Figure 1:**
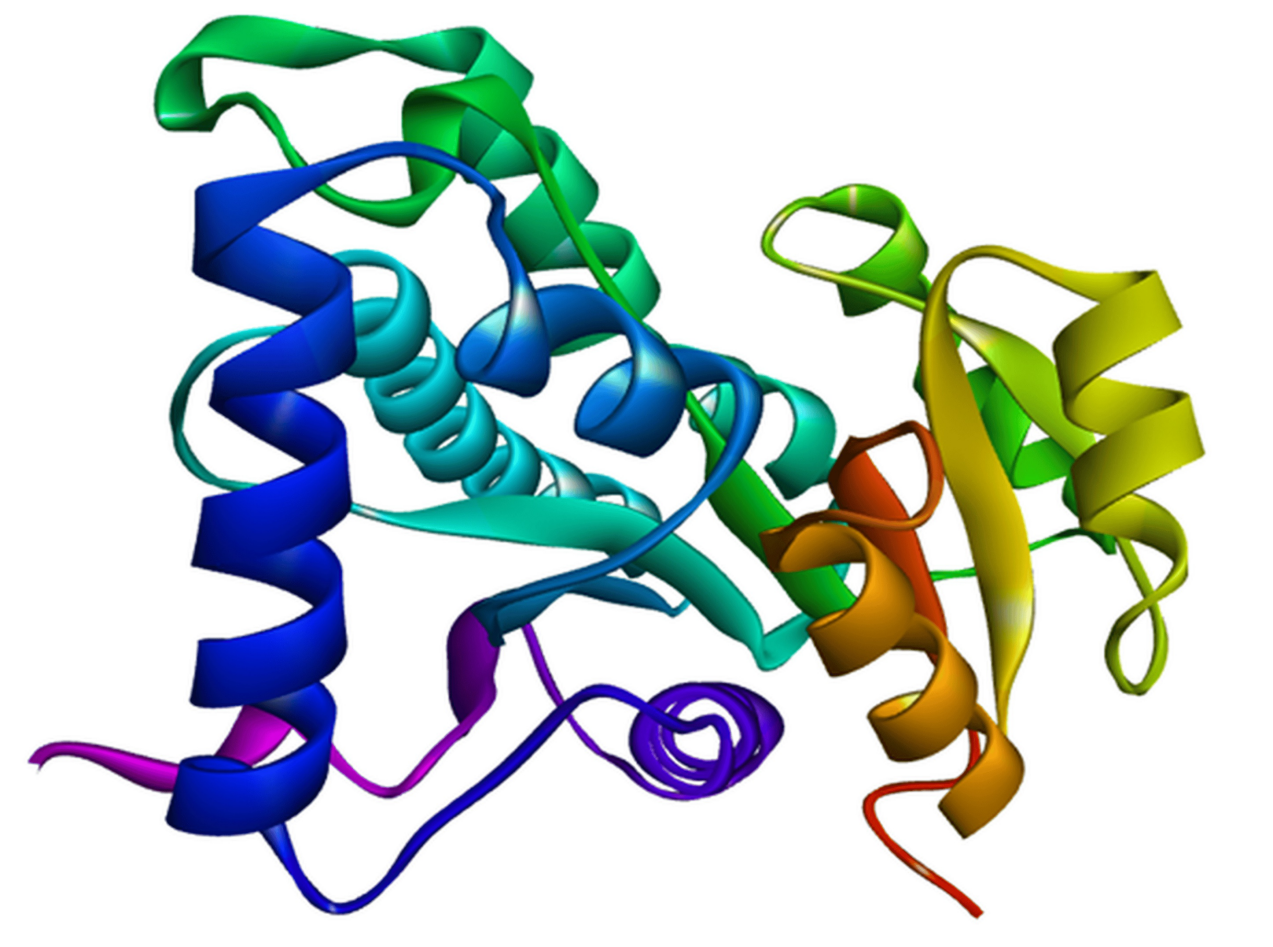
Prepared protein of 4U0J.

The protein crystal structure was downloaded from the Protein Database RCSB (PDB ID: 4U0J) and accessed through the website https://www.rcsb.org. The structure of 4U0J was determined at a high resolution of 1.6 Å. High-resolution structures provide accurate details of the protein's active site, which is crucial for reliable docking studies [23]. The Discovery Studio Visualizer software was used to prepare crystal complexes. In DS, a protein protocol was utilized to establish a consistent nomenclature for atoms, rectify connection and binding order, protonate ionizable residues at a pH of 7.4, insert missing residues, rectify incomplete ones, and eliminate water molecules. To validate the docking method, the ligand that was co-crystallized with the enzyme was extracted and subsequently re-docked into the active site. This was done to confirm that the binding site is accurately defined and to evaluate the docking algorithm's ability to reproduce the position of the co-crystallized ligand (Figure 1) [24]. The selection of 4U0J was driven by its scientific significance in studying the interactions between oxadiazole derivatives and InhA inhibitors [25]. We selected 4U0J over other structures (such as 2NV6 and 1ZID) due to its high resolution and the completeness of the active site details. This choice was made to ensure that our docking studies were based on the most accurate and detailed structural information available. The selection allowed for the analysis of the binding affinities' functional and structural characteristics and yielded valuable data for the future verification of the synthesized oxadiazole analogs through experimental and animal studies. The ligand-protein interaction enabled the discovery of the active regions within the protein. The active sites of the protein amino acids were Gly A:96, Ser A:94, Phe A:147, Met A:199, Ile A:21, and Thr A:196. These sites engage in several sorts of bonding, such as typical hydrogen bonds, pi-donor hydrogen bonds, pi-alkyl, pi-sigma, and pi-pi stacking interactions.

Computation of ADMET (absorption, distribution, metabolism, excretion, and toxicity) in silico

Novel drugs for research can be developed more efficiently and precisely due to computer technology and drug discovery developments. To better understand the ADMET of drugs, in silico research has become an essential tool. This method is used to conduct initial evaluations using pharmacokinetic metrics and pharmacological similarities during drug discovery. For instance, ADMET pharmacokinetic parameter evaluation in pharmaceutical research depends significantly on computer technology. By employing the web-based pkCSM software (Dr. David B. Ascher, Monash University, Melbourne, Australia) [26], we could evaluate a compound's potential for intestinal absorption, systemic distribution, metabolic transformation, elimination pathways, and toxicity levels.

Anti-TB activity using Alamar Blue dye assay

The synthesized compounds 3a-3e have been tested for their antimycobacterial activity toward TB strain (H37RV strain) utilizing the microplate Alamar Blue assay (MABA). This technology is distinguished by its lack of toxicity, use of thermally resistant reagents, and its close relationship with the volumetric and BACTEC radiometric procedures; 200 μL of sterilized deionized water was added to the outside boundary wells of a sterilized 96-well plate, and 100 μL of the Middlebrook 7H9 broth was added to each well. Synthesized compounds were diluted directly on the plate. Drug concentrations ranging from 100 to 0.2 μg/mL were selected for the study. The plates were securely enveloped and hermetically sealed with parafilm prior to being positioned in an incubation chamber adjusted to an ambient temperature of 37°C for a duration of five days. Following this time frame, a 25 μL quantity of a newly prepared solution containing Almar Blue and 10% Tween 80 in a 1:1 ratio was introduced to the plates and left to incubate for 24 hours. The existence of bacterial multiplication was revealed by a pink hue in the well plate, whereas the absence of bacterial proliferation was demonstrated by a blue hue. The minimum inhibitory concentration (MIC) was obtained by identifying the medication concentration at which the change of color from blue to pink was inhibited.

## Results

Chemistry

**Figure 2:**
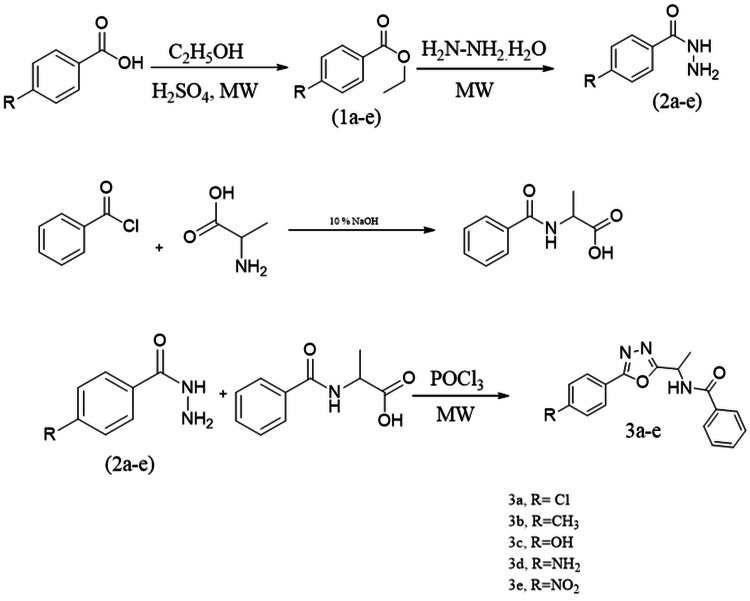
Synthetic scheme of N-[1-(5-phenyl-1,3,4-oxadiazol-2-yl) ethyl] benzamide (3a-3e).

The current study presents the synthesis of a novel group of N-[1-(5-phenyl-1,3,4-oxadiazol-2-yl) ethyl] benzamide derivatives, and the reaction involved in the synthetic process is outlined in Figure 2. Firstly, p-substituted aromatic acid was esterified in the presence of ethanol and sulfuric acid to create the ester derivatives, which in turn were subjected to hydrazinolysis in microwave irradiation to form an acid hydrazide derivative (2a-e). Secondly, the N-protected amino acids were synthesized by using benzoyl chloride and alanine to protect the -NH2 group, then the N-protected amino acid reacted with the acid hydrazide derivative in the presence of POCl3 to form 1,3,4-oxadiazole derivatives (3a-3e) using microwave conditions. The completion of the reaction was monitored by TLC and UV (Table 1). Microwave irradiation offers several benefits compared to conventional heating techniques, including enhanced reaction rate, increased chemical yield, decreased energy usage, a wide range of selectivity parameters, and targeted heating [27,28]. The acid hydrazide derivatives and amino acids mixture in POCl3 underwent microwave irradiation at 80°C, leading to a significant yield of the product, 3a-3e, in a shorter reaction time. The salient feature of this reaction is that the desired product was produced with a substantial yield and without the necessity for column purification. The resultant compounds were purified by recrystallization using methanol, and the melting point of the resultant compound was recorded (Table 1). The structural characterization of oxadiazole derivatives (3a-3e) was accomplished using a variety of spectroscopic techniques, including IR, NMR, and GC-MS spectroscopy analysis. The IR spectra revealed stretching bands at around 3217 cm^-1^ for -NH, 1695 cm^-1^ for C=O, and 1552 cm^-1^ for C=N, all linked with the arylidene hydrazide structure. The ^1^H NMR analysis revealed significant doublets at the chemical shifts of 3.93, indicating the presence of methyl proton next to amide and multiplets at d7.47-8.34, which can be attributed to aromatic protons in the benzamide derivatives (3a-3e). In the mass spectrum, the moderate-strength molecular ions are usually the acylium ions (m/z = 113), whereas the most potent peak typically corresponds to the nitrile radical ions (m/z = 55). These ions are produced via the fragmentation of heteroatom rings. Both of these qualities have been recorded as distinctive attributes of this family. These results suggest the successful synthesis of oxadiazole derivatives.

**Table 1:** The physical parameters, UV analysis, and TLC values of oxadiazole compounds CHCl_3_ - chloroform, DMSO - dimethyl sulfoxide, MeOH - methanol, TLC - thin-layer chromatography

Compounds	Structural formula	Molecular weight (g/mol)	Solubility	λ_max_	R_f _value
3a	C_17_H_14_ClN_3_O_2_	327.76	DMSO, MeOH, CHCl_3_	256	0.52
3b	C_18_H_17_N_3_O_2_	307.34	DMSO, MeOH, CHCl_3_	245	0.51
3c	C_17_H_15_N_3_O_3_	309.31	DMSO, MeOH, CHCl_3_	245	0.47
3d	C_17_H_14_N_4_O_4_	338.31	DMSO, MeOH, CHCl_3_	287	0.51
3e	C_17_H_16_N_4_O_2_	308.33	DMSO, MeOH, CHCl_3_	264	0.46

Molecular docking study

The synthesized compounds 3a-3e performed virtual screening (docking study) to determine their interaction with the selected target mycobacterial enoyl acyl carrier enzyme reductase (InhA) in Mtb. InhA plays a pivotal role in the synthesis of mycolic acids, which are vital components of the mycobacterial cell wall. Thus, the disruption of this pathway compromises the integrity of the cell wall, leading to bacterial cell death. In addition, intriguingly, we were enthused by the experimental MIC findings, which demonstrate a high degree of agreement with the inhibitory effects of the newly synthesized oxadiazole derivatives 3a-3e on InhA.

Examining the binding relationships between Mtb enoyl reductase (InhA) and its synthetic inhibitors

**Figure 3:**
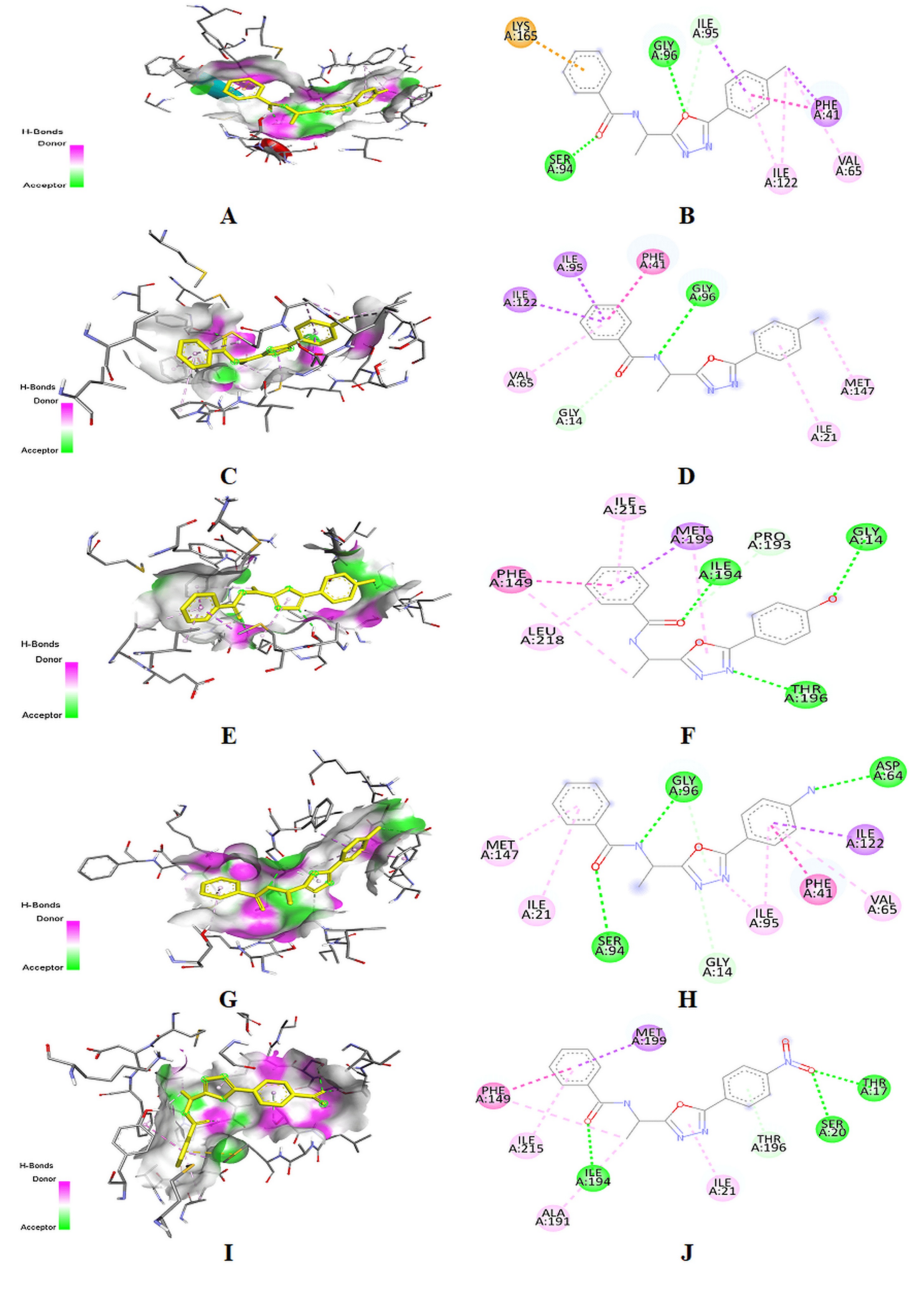
Crystal structure of InHA inhibitor (Protein Data Bank (PDB) ID: 4U0J) and binding of synthesized compounds (3a-3e). (A) Three-dimensional structure of 4U0J and binding of synthesized compound 3a. (B) Proposed binding amino acid and type of interaction are shown in different colors. (C) Three-dimensional structure of 4U0J and binding of synthesized compound 3b. (D) Proposed binding amino acid and type of interaction are shown in different colors. (E) Three-dimensional structure of 4U0J and binding of synthesized compound 3c. (F) proposed binding amino acid and type of interaction are shown in different colors. (G) Three-dimensional structure of 4U0J and binding of synthesized compound 3d. (H) proposed binding amino acid and type of interaction are shown in different colors. (I) Three-dimensional structure of 4U0J and binding of synthesized compound 3e. (J) proposed binding amino acid and type of interaction are shown in different colors.

The synthesized compounds underwent molecular docking utilizing the AutoDock Vina docking program with the 4U0J protein. The docking data yielded valuable information regarding the molecular interactions and binding strength between the ligands and their receptor, including the durability and ease of accessing these interactions. The compounds have exhibited binding affinities ranging from -8.7 to -9.1 kcal/mol, as indicated in Table 2. The compound 3d and the protein 4U0J consistently displayed a binding energy of -9.1 kcal/mol during their interactions, as illustrated in Figure 3. Compounds 3b, 3c, and 3d exhibited notable binding energies of -9.0, -9.0, and -9.1 kcal/mol, respectively, as shown in Table 2 and Figure 3. The ligand-protein complex interactions enabled the identification of active regions within the protein target. The ligand-protein complex exhibited diverse bonding interactions, including proton and polar bonds, acceptor-acceptor and donor-donor interactions, pi-sigma and pi-pi stacking interactions, and pi-alkyl interactions. The protein's active sites consist of specific amino acid residues, including glycine (Gly) 96, aspartic acid (Asp) 64, serine (Ser) 94, phenylalanine (Phe) 41, isoleucine (Ile) 95, Ile 122, and Gly 14. The synthesized compounds demonstrated a greater affinity for receptor binding compared to the reference medication, isoniazid (-5.6 kcal/mol). On the other hand, compound 3d exhibited the highest affinity for binding to the target protein, with a binding affinity of -9.1 kcal/mol. In addition, molecules 3a, 3b, 3d, and 3e, which exhibited the highest affinity toward the target, also establish hydrogen bonds with Ser 94. In addition, Figure 3 illustrates the three-dimensional interactions of the ligand with the active site of the protein.

**Table 2:** Docking data of synthesized substances against InhA (4U0J) Ala - alanine, Asp - aspartic acid, Gly - glycine, Ile - isoleucine, Leu - leucine, Lys - lysine, Met - methionine, Phe - phenylalanine, Pro - proline, Ser - serine, Thr - threonine, Val - valine

S. no.	Synthesized compounds	Binding affinity (kcal/mol)	Hydrogen bond interaction (amino acid)	Residual interaction (amino acids)
1	3a	-8.9	Ser 94, Gly 96	Lys 165, Ile 95, Phe 41, Ile 122, Val 65
2	3b	-9.0	Gly 96	Val 65, Gly 14, Ile 122, Ile 95, Phe 41, Met 147, Ile 21
3	3c	-9.0	Gly 14, Thr 196, Ile 194	Phe 149, Leu 218, Ile 215, Met 199, Pro 193
4	3d	-9.1	Gly 96, Ser 94, Asp 64	Met 147, Ile 21, Gly 14, Ile 95, Phe 41, Val 65, Ile 122
5	3e	-8.7	Ile 194, Ser 20, Thr 17	Met 199, Phe 149, Ile 215, Ala 191, Ile 21, Thr 196

In silico ADMET properties

Oxadiazole derivatives 3a-3e compounds against InhA proteins were tested using ADMET pharmacokinetic criteria [29]. The predicted outcomes obtained using pkCSM, a web-based tool, are presented in Table 3. It generally indicates insufficient absorption when the absorption percentage falls below 30%. Nevertheless, the findings revealed that all the substances exhibited favorable absorption in the human gastrointestinal tract. A low volume of distribution (VDss) is indicated by a logVDss value of less than -0.15, while a high volume of distribution is indicated by a logVDss value greater than 0.45. Average permeability values for the central nervous system (CNS) and blood-brain barrier (BBB) ranged from greater than -2 to less than -3 in terms of LogPS value and greater than 0.3 to less than -1 for LogBB. LogBB values below -1 indicate inadequate distribution to the brain, while LogBB values above 0.3 reflect the ability to pass the BBB. A LogPS value more than -2 is thought to have the ability to pass through the CNS, but a LogPS value greater than -3 is considered to have challenges in entering the CNS. Therefore, ligand 3c has a higher capacity to traverse barriers. Drug metabolism is the process of pharmacological compounds undergoing biological alteration within the body. Drugs undergo a series of enzymatic reactions, producing several metabolites with diverse qualities related to their effects, distribution in the body, and physical and chemical characteristics. Thus, understanding the drug metabolism process and the possibility of medication interactions is necessary for the identification of effective drug candidates biologically. Particularly inhibiting CYP1A2, 2C19, 2C9, 2D6, and 3A4 cytochrome P450 enzymes has an enormous impact on how drugs are metabolized and can cause drug-drug interactions. Therapeutic targeting can be directed toward these cytochrome P450 enzymes. Therefore, we have illustrated the metabolic process of each ligand, as outlined in Table 3. Of all the cytochromes, CYP1A2 was the most notable inhibitor [30]. Additionally, a decreased total clearance indicates that the drug remains in the body for a prolonged duration, which can be advantageous for the drug discovery process [31]. Ultimately, the findings of this investigation unequivocally verified that all the substances did not show any signs of toxicity (Table 3), a crucial prerequisite in developing new entities. Based on the data, it is possible to conclude that the in silico pharmacokinetic predictions produced positive results. The synthesized compounds (3a-3e) demonstrate exceptional kinetic characteristics, meet the required drug-likeness standards, and demonstrate noteworthy biological efficacy.

**Table 3:** Prediction of probable inhibitors using in silico ADMET Yes means inhibiting respective CYP isoenzymes. No indicates no inhibitory activity toward substrates. ADMET - absorption, distribution, metabolism, excretion, and toxicity, BBB - blood-brain barrier, CNS - central nervous system, CYP - cytochrome P450, PS - permeability surface area, VDss - volume of distribution

Compounds	Absorption	Distribution			Metabolism							Excretion	Toxicity
	Intestinal absorption (human)	VDss (Human)	BBB permeability	CNS permeability	Substrate		Inhibitor					Total clearance	Ames toxicity
					CYP								
	Numeric (% absorbed)	Numeric (LogL/kg)	Numeric (LogBB)	Numeric (LogPS)	2D6	3A4	1A2	2C19	2C9	2D6	34A	Numeric (LogmL/min/kg)	Categorical (yes/no)
					Categorical (yes/no)								
3a	91.226	-0.108	-0.417	-2.019	No	Yes	Yes	Yes	Yes	No	No	0.234	No
3b	92.684	-0.081	-0.241	-2.059	No	Yes	Yes	Yes	Yes	No	No	0.232	No
3c	90.623	-0.058	-0.334	-2.324	No	No	Yes	No	Yes	No	No	0.202	No
3d	92.141	-0.135	-0.286	-2.295	No	No	Yes	No	No	No	No	0.228	No
3e	88.483	-0.321	-0.321	-2.31	No	Yes	Yes	Yes	Yes	No	No	0.253	No

MABA assay - antitubercular activity

**Figure 4:**
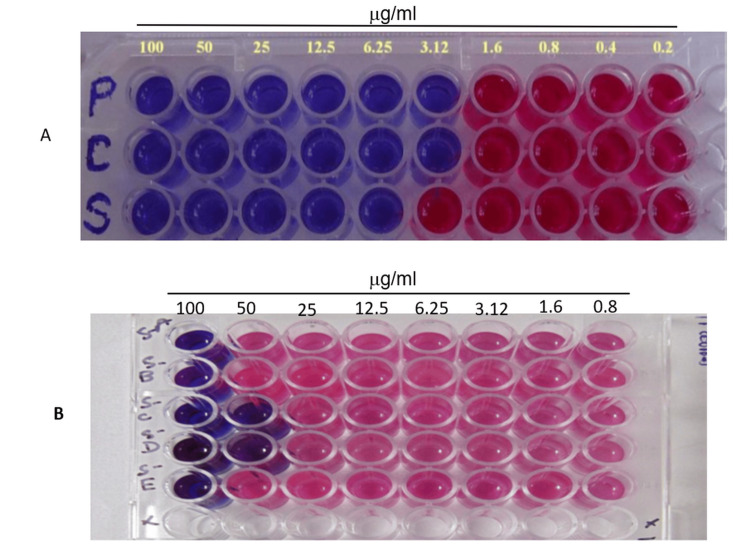
(A) Investigation of the antimycobacterial activity of conventional chemical compounds. P represented pyrazinamide and demonstrated antimycobacterial activity within the concentration range of 100-3.125 μg/mL. C represented ciprofloxacin and exhibited antimycobacterial activity within the concentration range of 100-3.125 μg/mL. S represented streptomycin and displayed antimycobacterial activity within the 100-6.25 μg/mL concentration range. The presence of the blue color signifies the lack of bacterial proliferation. (B) Antimycobacterial activity of the synthesized compounds (3a-3e) at a concentration of 100-0.8 μg/mL. The blue color indicates the absence of bacterial growth.

It was examined using the MABA method to determine whether the synthesized derivatives 3a-3e inhibited the Mtb (H37Rv). The concentrations of standard drugs were utilized within the range of 100 μg/mL to 0.2 mg/mL (Figure 4A). The antitubercular activity of the synthesized compounds was measured in the lab using the MABA method to determine its MIC (µg/mL). The well's blue color indicated that there were no bacterial growths present. However, the pink hue stated that there had been growth. Finding the drug concentration at which the color transitioned from blue to pink was considered the MIC (Figure 4B). The assay results for the synthesized compounds 3a-3e to determine their MIC are displayed in Table 4 (μg/mL). MIC at a concentration of 50 μg/mL and derivatives 3c and 3d were found to have the most activity out of all the compounds. Some compounds 3a, 3b, and 3e were slightly effective against Mtb at 100 μg/mL concentrations (Figure 4B).

**Table 4:** The minimum inhibitory concentration of in vitro anti-tubercular activity of synthesized compounds 3a-3e S - sensitive, R - resistance

Compounds	100 μg/mL	50 μg/mL	25 μg/mL	12.5 μg/mL	6.25 μg/mL	3.12 μg/mL	1.6 μg/mL	0.8 μg/mL
3a	S	R	R	R	R	R	R	R
3b	S	R	R	R	R	R	R	R
3c	S	S	R	R	R	R	R	R
3d	S	S	R	R	R	R	R	R
3e	S	R	R	R	R	R	R	R
Standard								
Pyrazinamide	S	S	S	S	S	S	R	R
Ciprofloxacin	S	S	S	S	S	S	R	R
Streptomycin	S	S	S	S	S	R	R	R

## Discussion

In this study, we successfully synthesized a novel series of N-[1-(5-phenyl-1,3,4-oxadiazol-2-yl) ethyl] benzamide derivatives and conducted a comprehensive analysis of their chemical structure and biological activity. The synthesis was achieved using a microwave-assisted approach, which offers several advantages over conventional methods, including shorter reaction times, higher yields, and reduced energy consumption. The absence of the need for column purification further highlights the efficiency of the method, making it a potentially valuable approach for synthesizing similar compounds.

The structural characterization of the synthesized oxadiazole derivatives (3a-3e) was confirmed through various spectroscopic techniques, including IR, NMR, and GC-MS. The IR spectra showed characteristic absorption bands corresponding to the key functional groups, while the ^1^H NMR spectra provided detailed insights into the chemical environment of the protons within the molecules. The GC-MS analysis further supported the successful synthesis by providing molecular ion peaks consistent with the expected structures. These results collectively confirm the successful synthesis and purity of the target compounds.

The molecular docking study provided insights into the potential biological activity of the synthesized compounds against the Mtb enoyl acyl carrier enzyme reductase (InhA). The docking results revealed strong binding affinities between the synthesized compounds and the InhA enzyme, with compound 3d showing the highest binding affinity of -9.1 kcal/mol. The docking interactions suggested that these compounds could effectively inhibit the InhA enzyme, which is crucial for the synthesis of mycolic acids in the bacterial cell wall. The inhibition of this enzyme could potentially lead to the disruption of the mycobacterial cell wall, resulting in bacterial cell death. The strong correlation between the docking results and the experimental MIC values further supports the potential of these compounds as effective antitubercular agents.

The in silico ADMET analysis provided valuable information on the pharmacokinetic properties of the synthesized compounds. The results indicated that the compounds possess favorable ADME profiles with no significant toxicity concerns. The compounds demonstrated good gastrointestinal absorption and a suitable distribution profile, with some compounds showing the potential to cross the BBB. The metabolic analysis revealed that the compounds are likely to undergo metabolism by cytochrome P450 enzymes, particularly CYP1A2, which could impact their pharmacokinetic behavior. The overall ADMET profile suggests that these compounds have the potential to be developed as drug candidates with favorable pharmacokinetic properties.

The antitubercular activity of the synthesized derivatives 3a-3e was evaluated using the MABA method against the Mtb H37Rv strain. The results revealed that compounds 3c and 3d exhibited significant activity with MIC of 50 μg/mL, indicating their potential as antitubercular agents. When compared to recent studies, these findings align well with other potent compounds reported in the literature. For instance, Bromo-pyridyl tethered 3-chloro 2-azetidinone derivatives exhibited MIC values ranging from 12.5-100 μg/mL, with the most potent compound having an MIC of 100 μg/mL [32]. Similarly, coumarin-based derivatives showed MIC values between 25 and 100 μg/mL, with the most active compound exhibiting an MIC of 25 μg/mL [33]. Additionally, 1,3,4-oxadiazole derivatives also displayed a comparable range of activity, with MIC values ranging from 12.5 to 100 μg/mL [34]. These comparisons indicate that the synthesized compounds 3c and 3d, with their MIC of 50 μg/mL, are within the range of effectiveness observed in other recently studied compounds. In contrast, the other synthesized compounds, 3a, 3b, and 3e, which exhibited MICs of 100 μg/mL, demonstrate moderate activity but are still within the spectrum of antitubercular efficacy reported in contemporary research.

## Conclusions

Multiple antimycobacterial drugs are now accessible, but increasing instances of multi-drug resistance constrain their efficacy. Thus, to develop novel medications to treat TB, scientists are focusing on identifying particular molecules that can be targeted and comprehending how they function. The present research involves the synthesis of a novel set of five compounds, specifically oxadiazole derivatives 3a-3e, using the microwave-assisted approach, resulting in a high yield. Multiple analytical spectroscopic techniques were employed to determine the structure of the material. All the synthesized compounds exhibited significant antimycobacterial activity against the Mtb H37RV strain. However, compounds 3c and 3d demonstrated the highest inhibitory effect. The estimated ADMET parameters confirm the presence of favorable pharmacokinetic features. Docking analysis determined how strongly the substances were bonded to InhA, an enzyme inhibiting enoyl-ACP in the Mtb. All compounds were docked into the same region for this purpose. Compound 3d forms the most energetically favorable bonds with a binding energy of -9.1 kcal/mol.
